# Metal-Free Nitrogen-Doped Carbon Derived from g-C_3_N_4_/Glucose Composites for Singlet Oxygen-Dominated PMS Activation Toward Enrofloxacin Degradation

**DOI:** 10.3390/nano16211427

**Published:** 2026-11-05

**Authors:** Peilun Wang, Lei Wang, Xinyu Zhang, Yinlong Kuang, Shuming Liu, Le Cui, Yixue Xu, Qiufang Yao

**Affiliations:** 1 College of Advanced Materials Engineering, Jiaxing Nanhu University, Jiaxing 314001, China; 2 Yangtze River Delta Institute of Circular Economy Technology, Jiaxing 314001, China; 3 Shanghai Chengtou Water Group Co., Ltd., Shanghai 200092, China; 4 College of Civil Engineering and Architecture, Zhejiang University, Hangzhou 310058, China

**Keywords:** nitrogen-doped carbon, peroxymonosulfate, singlet oxygen, enrofloxacin, metal-free catalyst, non-radical pathway

## Abstract

Enrofloxacin (ENR), a widely used veterinary fluoroquinolone, persists in the environment and resists conventional treatment. A metal-free nitrogen-doped carbon catalyst (N@C) was synthesized from graphitic carbon nitride (g-C_3_N_4_) and glucose via hydrothermal compositing and carbonization at 900 °C, and used to activate peroxymonosulfate (PMS) for ENR degradation. TEM, XRD, XPS, Raman spectroscopy, and N_2_ physisorption revealed a hierarchically porous architecture with a specific surface area of 578.6 m^2^ g^−1^ and 10.2 at% nitrogen. The N@C/PMS system removed over 99.9% of ENR in 20 min ([ENR]_0_ = 20 mg L^−1^, [PMS] = 0.2 mM, [N@C] = 5 mg L^−1^, 25 °C), with a rate constant of 0.4389 min^−1^. TOC removal reached 40% within 30 min at 1.0 mM PMS, and post-reaction XPS and XRD showed a preserved framework. Quenching tests, DPA probing, solvent isotope effects, and electron spin resonance (ESR) spectroscopy indicate a non-radical pathway dominated by singlet oxygen (^1^O_2_), initiated by heterolytic cleavage of the PMS peroxide bond at graphitic N sites. The catalyst retained >95% of its activity after four cycles and >93% removal in tap, river, and lake water. These findings provide a mechanistic basis for designing metal-free, matrix-tolerant persulfate activation systems for fluoroquinolone wastewater treatment.

## 1. Introduction

Fluoroquinolone antibiotics are extensively used in human medicine, livestock husbandry, and aquaculture, yet a substantial fraction of the administered dose is excreted unmetabolized and enters receiving waters through wastewater effluents and surface runoff [1,2]. Enrofloxacin (ENR), a veterinary fluoroquinolone, is one of the most ubiquitously detected antibiotic residues in global surface water and sediment, with reported concentrations spanning the ng L^−1^ to low µg L^−1^ range [1,2,3]. Its environmental persistence stems from the innate stability of the fluorinated quinolone core, which resists hydrolysis and direct photolysis, while its amphoteric character promotes sorption to particulates and prolongs environmental residence [3]. Chronic exposure to sub-inhibitory ENR concentrations drives the propagation of antibiotic resistance genes (ARGs) and disruption of indigenous microbial communities [3]. Conventional biological and physicochemical treatment processes achieve only partial ENR removal, highlighting the need for alternative abatement technologies [4].

Peroxymonosulfate (PMS)-driven advanced oxidation processes (AOPs) have garnered extensive research interest owing to the facile activation of their asymmetric peroxide bond to produce powerful reactive oxidants; they also operate over a broad pH range and remain relatively tolerant of organic-rich matrices [5,6]. Developing efficient, durable, and eco-friendly PMS activators remains a core research target in this field [4,5,6]. However, several bottlenecks constrain the application of PMS-based technologies to real ENR-containing waters. Transition-metal and metal-oxide catalysts (Co, Fe, Cu, Mn and their bimetallic derivatives) deliver high activity but suffer from metal-ion leaching and associated ecotoxicity; cobalt-based systems in particular raise concerns over bioaccumulation that limit practical deployment [2,7,8,9]. Radical-dominated pathways driven by sulfate radical (SO_4_•^−^) and hydroxyl radical (•OH) are highly vulnerable to scavenging by ubiquitous matrix constituents such as bicarbonate, chloride, and natural organic matter, which competitively consume reactive species and can yield transformation products of comparable or elevated toxicity [6,10]. A structurally refractory pollutant such as ENR has a rigid quinolone ring and a fluorinated substituent that impose a high energy barrier to radical oxidation and thus suppress total mineralization; consequently, metal-free carbonaceous activation routes remain insufficiently explored, and existing materials rarely reconcile high activity with long-term stability and matrix tolerance [4,10].

In the specific context of nitrogen-doped carbon/PMS systems, two critical gaps persist. First, at the mechanistic level, the identity of the dominant active sites—graphitic N, pyridinic N, pyrrolic N, and topological defects—in PMS activation remains contested, and the generation pathway of singlet oxygen (^1^O_2_) together with the role of interfacial electron transfer are not systematically resolved; correspondingly, the ring-opening, defluorination, and mineralization routes of ENR under carbon-based non-radical conditions are poorly defined [10,11,12,13,14]. Second, at the materials and performance level, many carbon catalysts are limited by insufficient surface area, low active-site density, and poor intraparticle mass transfer, so that high degradation activity, multi-cycle stability, and resistance to background interference are rarely achieved simultaneously under realistic water matrices [4,13,15]. Most nitrogen-doped carbon catalysts are derived from graphene, biomass, or synthetic polymers; reports employing graphitic carbon nitride (g-C_3_N_4_) as a nitrogen-rich precursor to engineer carbon architectures for PMS activation remain limited. Zhang et al. [16] recently varied the nitrogen content of the g-C_3_N_4_/carbon composite and thereby the nitrogen speciation, graphitization, and defect structure, and identified graphitic and pyridinic N as the catalytic sites for levofloxacin degradation. The co-pyrolysis route is therefore no longer unexplored, and the present study does not claim it as such. What remains unresolved is how the individual nitrogen sites steer the reactive-oxygen pathway.

To address these challenges, this study develops a metal-free nitrogen-doped carbon catalyst (denoted N@C) synthesized from g-C_3_N_4_ and glucose through a hydrothermal–carbonization route. The design rationale exploits the nitrogen-rich g-C_3_N_4_ framework and its intimate compositing with the glucose-derived carbon phase; upon pyrolysis, this yields a carbon architecture with high nitrogen content, abundant topological defects, hierarchical micro–mesoporosity, and a large specific surface area, providing a high density of PMS activation sites and favorable mass-transfer pathways [1,11,13,14]. Elevated nitrogen doping strengthens π-electron delocalization and the electron-donating capacity of the carbon skeleton, promoting heterolytic cleavage of the PMS O–O bond [11]. The specific objectives of this work are to: (1) fabricate and systematically characterize N@C with emphasis on nitrogen speciation and defect structure; (2) evaluate its catalytic performance for ENR degradation under various operational conditions, including cycling stability and applicability in authentic water matrices; (3) elucidate the reactive species and non-radical mechanism through radical quenching, electron spin resonance (ESR), and X-ray photoelectron spectroscopy (XPS) analyses; and (4) establish the structure–activity relationship governing ^1^O_2_ generation in this metal-free system.

Three specific questions are addressed here. (i) Is ^1^O_2_ generated downstream of a superoxide (•O_2_^−^) relay, as proposed for the related g-C_3_N_4_@glucose system [16], or does it form by direct heterolytic cleavage of the O–O bond of a surface-activated PMS complex? (ii) Do graphitic N and pyridinic N act as equivalent activation centers, or is there a division of labor between them? (iii) Which descriptor controls ^1^O_2_ productivity: the total nitrogen content, the absolute abundance of a single nitrogen species, or the relative population of the two configurations? The third question cannot be settled by a single material, and is therefore formulated as a testable descriptor to be evaluated across a series of catalysts (Section 4).

Finally, the choice of a dark catalytic PMS process rather than a heterojunction photocatalytic one is deliberate, because the two routes address different constraints. Secondary effluents from livestock and aquaculture operations are turbid and coloured, so photon penetration is limited, and a dark catalytic step requires comparatively little modification of existing tanks. The two routes are nevertheless convergent rather than competing: because N@C is a metal-free carbon with a delocalized π-system, it can act as an electron mediator or co-catalyst when combined with a semiconductor light absorber, coupling photocatalytic charge separation with PMS activation in a single material.

## 2. Materials and Methods

### 2.1. Chemicals and Reagents

All chemicals were of analytical or higher grade and used without further purification. Ultrapure water was used throughout. Potassium peroxymonosulfate (PMS, 42.8–46% purity) and enrofloxacin (ENR, ≥98%) were obtained from Adamas Reagent Co., Ltd. (Shanghai, China) and Shanghai Yuanye BioTech Co., Ltd. (Shanghai, China), respectively. Radical scavengers including methanol (HPLC grade), tert-butanol (TBA), dimethyl sulfoxide (DMSO), furfuryl alcohol (FFA), and L-histidine, along with spin-trapping agents 5,5-dimethyl-1-pyrroline N-oxide (DMPO) and 2,2,6,6-tetramethylpiperidine (TEMP), were sourced from Adamas or Aladdin Biochemical Technology Co., Ltd. (Shanghai, China). The precursors for catalyst synthesis, urea and glucose (analytical grade), were supplied by Aladdin. Deionized (DI) water was obtained by purifying Millipore ultrapure water (resistivity = 18.2 MΩ·cm, 298 K).

### 2.2. Synthesis of g-C_3_N_4_ Precursor

Urea (analytical reagent) was placed in a muffle furnace (SX2-5-12, Yiheng, Shanghai, China) and directly calcined at 550 °C for 4 h under N_2_ atmosphere. After natural cooling to room temperature, the pale-yellow product was collected and ground into a fine powder for subsequent use.

### 2.3. Preparation of N@C

Typically, 0.5 g of the as-prepared g-C_3_N_4_ was dispersed in 40 mL of an aqueous glucose solution (0.4 M) inside a 100 mL Teflon-lined stainless-steel autoclave. The suspension was stirred for 3 h and then sealed, followed by hydrothermal treatment at 140 °C for 3 h in an oven. After natural cooling, the g-C_3_N_4_/carbon composite was collected by centrifugation, washed sequentially with ultrapure water and ethanol, and dried at 60 °C for 12 h. The dried precursor was transferred to a tube furnace (GSL-1500X-OTF, Kejing, Hefei, China) and calcined at 900 °C for 1 h under a continuous argon (Ar) flow at a heating rate of 5 °C min^−1^ to yield the nitrogen-doped carbon catalyst, denoted N@C.

### 2.4. Characterization

The physicochemical properties of the catalysts were characterized. Surface morphology and microstructure were examined by transmission electron microscopy (TEM, JEM 2100F, JEOL, Tokyo, Japan). Crystal phases were identified via X-ray diffraction (XRD, D8 Advance, Bruker, Karlsruhe, Germany) using Cu Kα radiation, while Raman spectra were recorded on a LabRAM HR Evolution spectrometer (Horiba, Kyoto, Japan) to probe carbon structural disorder. Specific surface area and pore structure were determined by nitrogen adsorption–desorption isotherms on a Micromeritics ASAP 2460 analyzer (Norcross, GA, USA) using the Brunauer–Emmett–Teller (BET) method. Surface chemical compositions and elemental bonding states were analyzed by X-ray photoelectron spectroscopy (XPS, ESCALAB 250 Xi, Thermo Fisher Scientific, Waltham, MA, USA).

### 2.5. Degradation Experiments and Analytical Methods

Batch degradation experiments were conducted in a 250 mL Erlenmeyer flask placed in a thermostatic water bath (HH-S4, Changzhou, China) at 25 °C. In a typical run, a known amount of N@C catalyst was dispersed into an ENR solution (20 mg L^−1^), and the reaction was initiated by adding PMS (0.2 mM). Control systems (ENR alone, ENR + PMS without catalyst, ENR + catalyst without PMS) were operated under identical conditions to assess individual contributions. Unless otherwise stated, the standard experimental conditions were: [ENR]_0_ = 20 mg L^−1^; [PMS] = 0.2 mM; [N@C] = 5 mg L^−1^; T = 25 °C. At predetermined time intervals, aliquots of the reaction suspension were withdrawn and immediately filtered through a 0.22 µm nylon membrane (Millipore, Burlington, MA, USA) to terminate the catalytic reaction. The residual ENR concentration was quantified by high-performance liquid chromatography (HPLC, Agilent 1200, Agilent Technologies, Santa Clara, CA, USA) equipped with a Shim-pack VP-ODS column (250 × 4.6 mm, Shimadzu, Kyoto, Japan) and a fluorescence detector. The mobile phase consisted of a mixture of 0.1% formic acid aqueous solution and acetonitrile (80:20, *v*/*v*) at a flow rate of 1.0 mL min^−1^; the injection volume was 50 µL and the excitation and emission wavelengths were set at 280 and 450 nm, respectively. The column temperature was maintained at 35 °C. Mineralization was monitored by measuring the total organic carbon (TOC) of the filtered samples on a total organic carbon analyzer (Multi N/C 3100, Analytik Jena, Jena, Germany). Because the oxidant dose governs the extent of mineralization, the TOC runs were carried out at a higher PMS concentration than the standard runs, namely [PMS] = 1.0 mM, with [ENR]_0_ = 20 mg L^−1^, [N@C] = 5 mg L^−1^ and T = 25 °C for 30 min. A parallel control without PMS was run under otherwise identical conditions to quantify the contribution of adsorption.

### 2.6. Quenching Tests, Probe Experiments and ESR Spectroscopy

Quenching tests were performed by adding a specific scavenger prior to PMS addition under otherwise identical conditions: TBA (0.1 M, for •OH), MeOH (0.1 M, for •OH and SO_4_•^−^), FFA (0.02 M, for ^1^O_2_), L-histidine (0.02 M, for ^1^O_2_), and DMSO (0.1 M, for •O_2_^−^). Electron spin resonance (ESR) spectra were recorded on a Magnettech MiniScope MS 5000 spectrometer (Berlin, Germany). Singlet oxygen (^1^O_2_) was trapped with TEMP added to the aqueous reaction suspension, and •OH/SO_4_•^−^ with DMPO. A stoichiometric probe, 9,10-diphenylanthracene (DPA), was also examined; its consumption was followed by UV–vis spectrophotometry at 378 nm (Supplementary Text S1).

### 2.7. Cycling Stability and Reusability

After each degradation cycle, the catalyst was recovered by centrifugation, washed with ultrapure water and ethanol, dried at 60 °C for 12 h, and reintroduced into a fresh reaction solution for the next run. A total of four consecutive cycles were performed and the activity retention was recorded. The catalyst recovered after the fourth cycle was additionally characterized by XRD and XPS.

### 2.8. Applicability in Real Water Matrices

The degradation performance was further evaluated in natural water samples (river water, lake water, and tap water) collected from Hangtang River (West Zhonghuan Road Reach), South Lake (Jiaxing), and Jiaxing Municipal Tap Water. Samples were filtered through 0.22 µm membranes and used as received, with all other conditions kept identical to those in Section 2.5.

### 2.9. Data Processing

The ENR removal efficiency (R%) was calculated as R% = 100 × (C_0_ − C_t_)/C_0_, where C_0_ and C_t_ denote the ENR concentration at the initial state and at reaction time t, respectively. Pseudo-first-order kinetics were evaluated by fitting ln(C_0_/C_t_) = *k*_obs_ × t, where *k*_obs_ is the apparent rate constant (min^−1^). All experiments were conducted at least in triplicate, and data are reported as mean values.

## 3. Results and Discussion

### 3.1. Structural and Chemical Characterization of N@C

#### 3.1.1. Morphology and Microstructure

**Figure 1:**
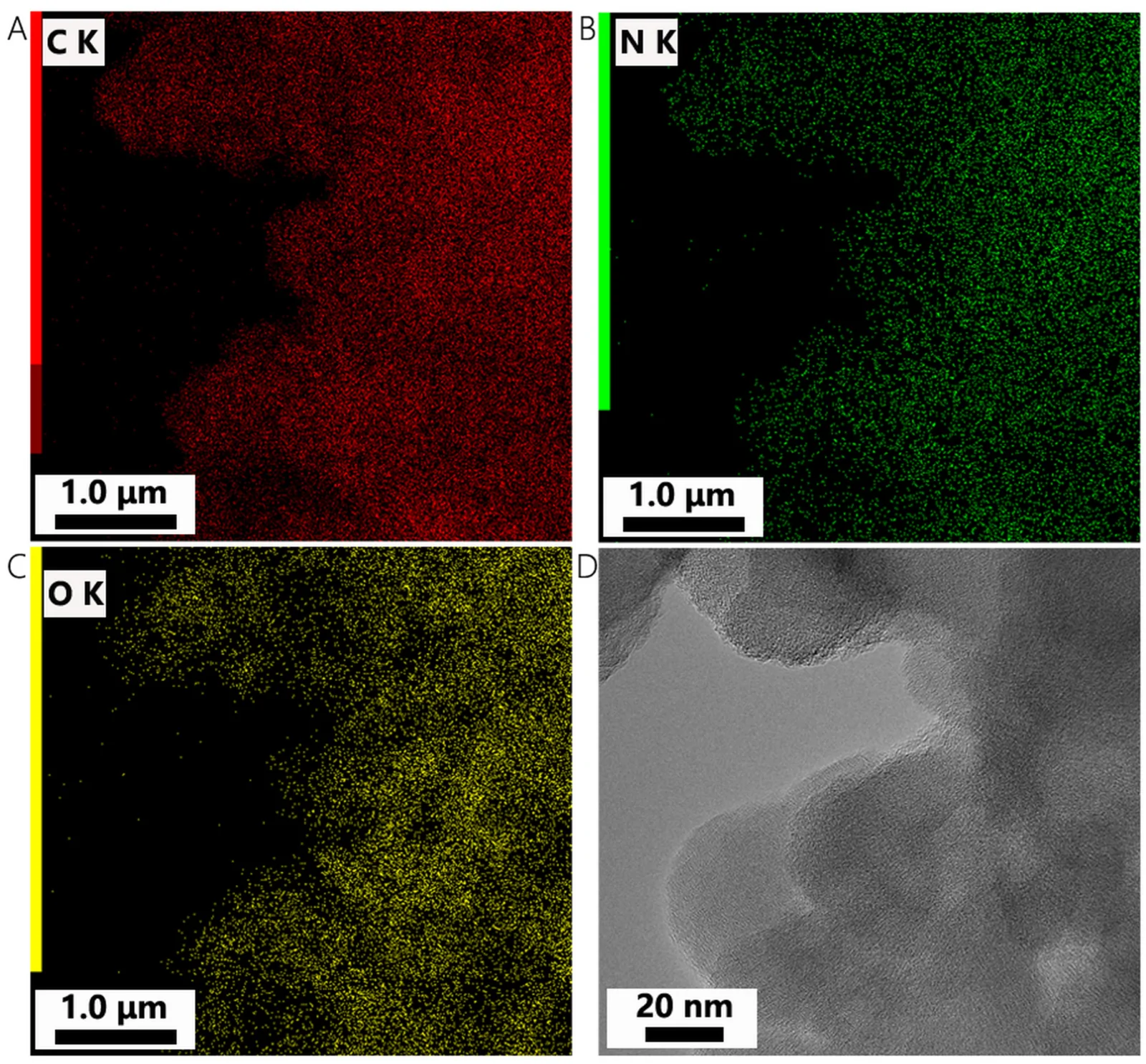
Elemental mapping images of N@C: C K (**A**), N K (**B**), O K (**C**), TEM image of N@C (**D**).

Figure 1A–C show STEM-EDS elemental mapping images of N@C, revealing the spatial distribution of carbon (C K), nitrogen (N K), and oxygen (O K), respectively. The uniform dispersion of N and O throughout the carbon framework confirms successful heteroatom doping during pyrolysis. Figure 1D shows a bright-field TEM image of N@C with a crumpled, sheet-like architecture and semi-transparent, folded nanosheet edges. Such crumpled and wrinkled nanosheet structures originate from the thermal exfoliation of bulk g-C_3_N_4_ precursor, and the few-layer thickness (a few nanometers) is evident from the transparency of the nanosheets. 

#### 3.1.2. Crystallinity and Defect Structure

**Figure 2:**
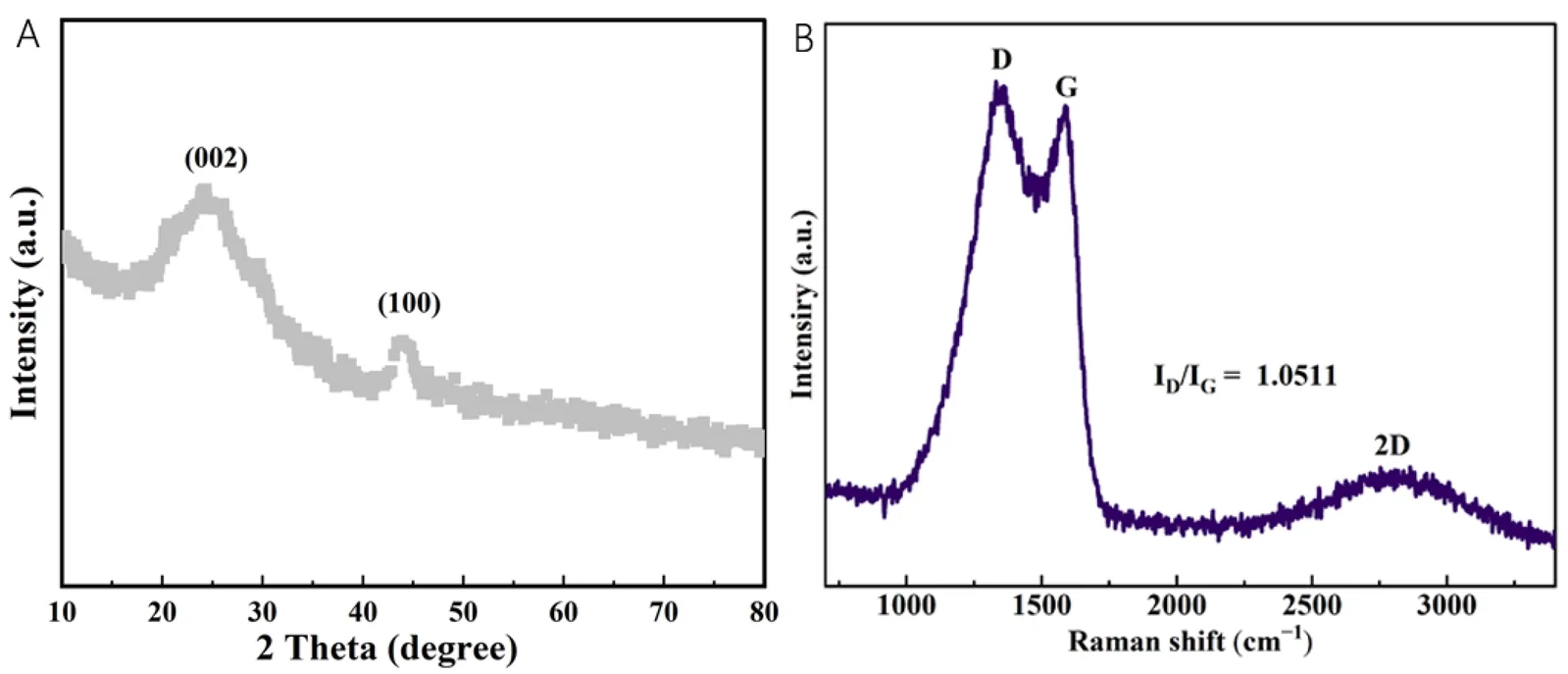
XRD pattern (**A**) and Raman spectrum (**B**) of N@C.

The crystallographic order of N@C was probed by powder XRD. The diffraction pattern (Figure 2A) displays only two broad, low-intensity reflections, consistent with the amorphous character observed by TEM image. The dominant peak at 2θ ≈ 24.5° corresponds to the (002) interlayer stacking of graphitic carbon with a d-spacing of ≈3.63 Å, while a weak shoulder near 44° is indexed to the in-plane (100) reflection of the hexagonal carbon network. The pronounced peak broadening—rather than the sharp peaks characteristic of crystalline graphite—indicates a turbostratic or amorphous product, in line with the substantial nitrogen content (10.2 at%, discussed below) that disrupts lattice periodicity through heteroatom substitution. Raman spectroscopy was employed to assess the degree of structural disorder. As displayed in Figure 2B, the spectrum exhibits the canonical D band (~1350 cm^−1^) and G band (~1580 cm^−1^), with a broad 2D band near 2700 cm^−1^ evidencing a layered, turbostratic carbon structure. The integrated intensity ratio I_D_/I_G_ was 1.0511, slightly exceeding unity. Values above 1.0 are characteristic of nitrogen-doped carbons obtained at high pyrolysis temperatures, where heteroatom substitution and vacancy formation generate a high density of catalytically relevant defects—vacancies, edge atoms, and grain boundaries—that lower the barrier for PMS chemisorption and act as secondary activation centers complementary to graphitic N [17,18,19,20].

#### 3.1.3. Surface Chemistry and Nitrogen Speciation

**Figure 3:**
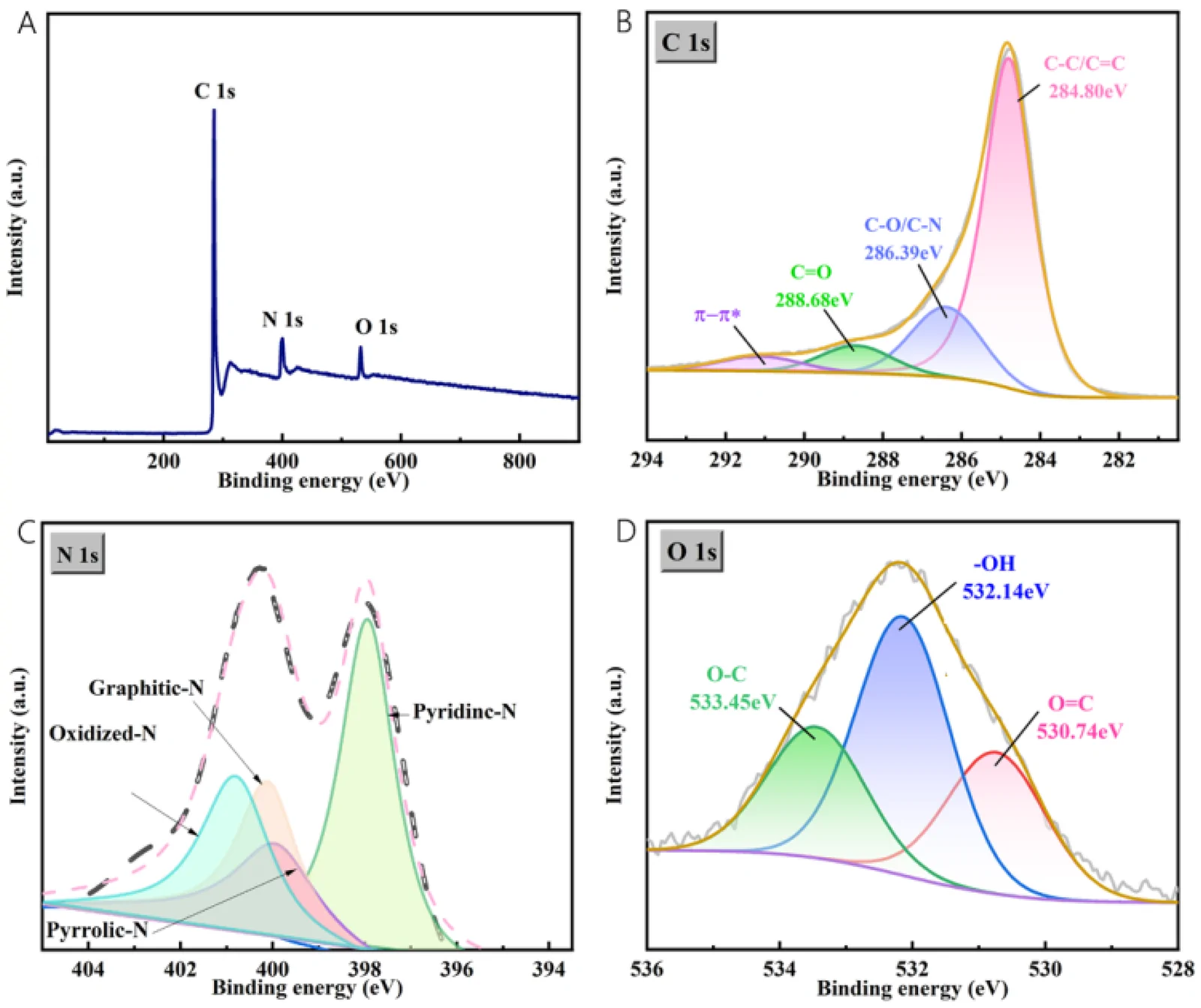
Full spectrum of XPS (**A**) and High-resolution XPS C 1s (**B**), N 1s (**C**), and O 1s (**D**) spectra of N@C.

Surface elemental composition and chemical states were determined by XPS. Survey scans (Figure 3A) identified C, N, and O as the only constituent elements, with no detectable metal signal, confirming the metal-free nature of the catalyst. The C 1s spectrum in Figure 3B was resolved into four components: sp^2^ C–C/C=C (284.80 eV), C–O/C–N (286.39 eV), C=O (288.68 eV) and a weak π–π* shake-up satellite (≈290.7 eV); the O 1s spectrum (Figure 3D) was deconvoluted into –OH (532.14 eV), O–C (533.45 eV) and O=C (530.74 eV) components. The high nitrogen content (10.2 at%) coexists with a defect-rich framework, consistent with the Raman results. Deconvolution of the N 1s spectrum (Figure 3C) yielded four components at 397.93 eV (pyridinic N), 399.20 eV (pyrrolic N), 400.05 eV (graphitic N) and 401.02 eV (oxidized N). The combined abundance of pyridinic and graphitic N exceeded that of pyrrolic N, a distribution favorable for PMS activation: graphitic N transfers electron density to its neighboring carbon atoms, generating electron-rich nucleophilic active sites for PMS adsorption and activation [17,21,22].

#### 3.1.4. Porosity

**Figure 4:**
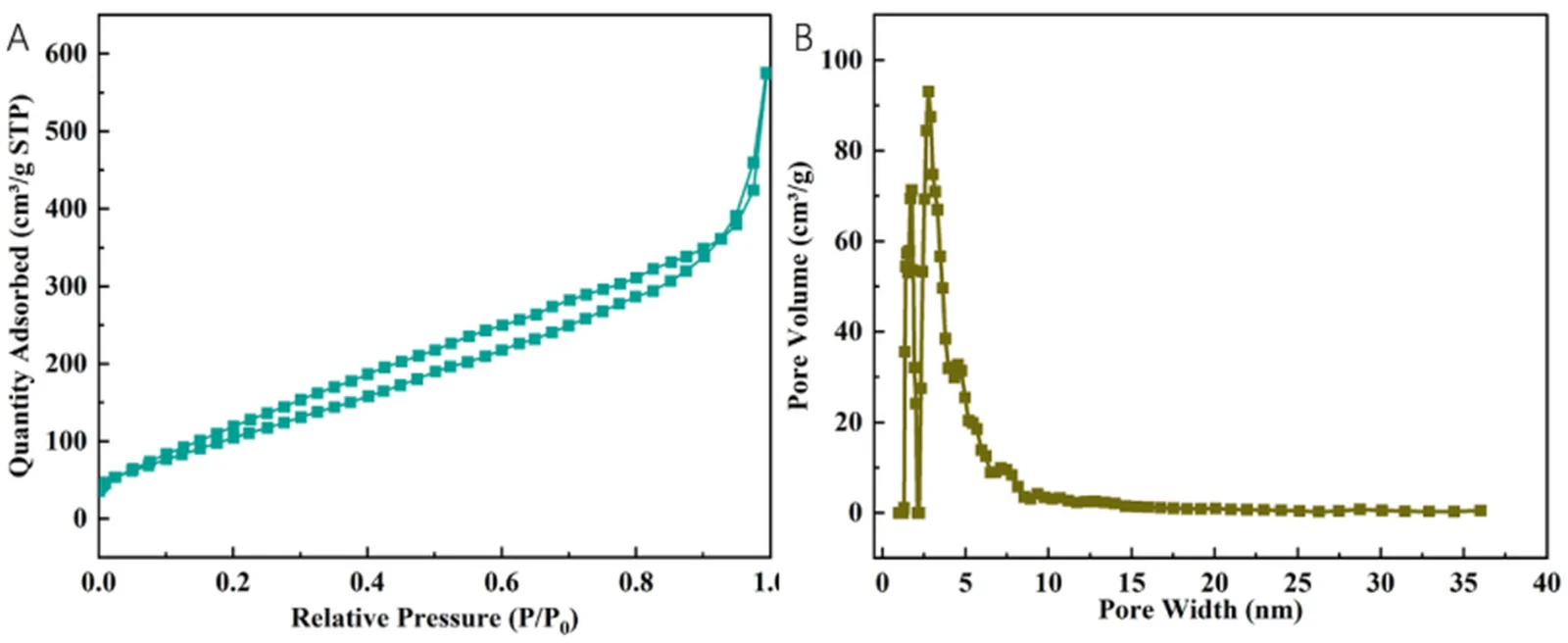
N_2_ adsorption–desorption isotherm (**A**); the BJH pore size distribution (**B**) of N@C.

N_2_ physisorption analysis (Figure 4) revealed a type IV isotherm with a prominent H4 hysteresis loop in the P/P_0_ range 0.4–1.0, characteristic of slit-shaped mesopores, while the steep uptake at P/P_0_ < 0.1 indicates coexisting micropores. The BET specific surface area reached 578.6 m^2^ g^−1^, substantially exceeding typical values for undoped carbonized glucose (~50–100 m^2^ g^−1^); this enhancement is attributed to the templating effect of g-C_3_N_4_ nanosheets and gas-released pore formation during pyrolysis. BJH analysis placed the mesopore size in a narrow 2–5 nm window. The combination of high surface area, micropores for active-site exposure, and mesopore channels for transport minimizes diffusional limitations during PMS activation. Collectively, the characterization establishes N@C as a hierarchically porous, nitrogen-rich (10.2 at%), and defect-abundant metal-free carbon whose structural attributes provide an excellent platform for the catalytic performance evaluated below [17,18,23].

### 3.2. Catalytic Degradation Performance

#### 3.2.1. Effect of Initial ENR Concentration, Catalyst and PMS Dosages; Catalytic Activity

**Figure 5:**
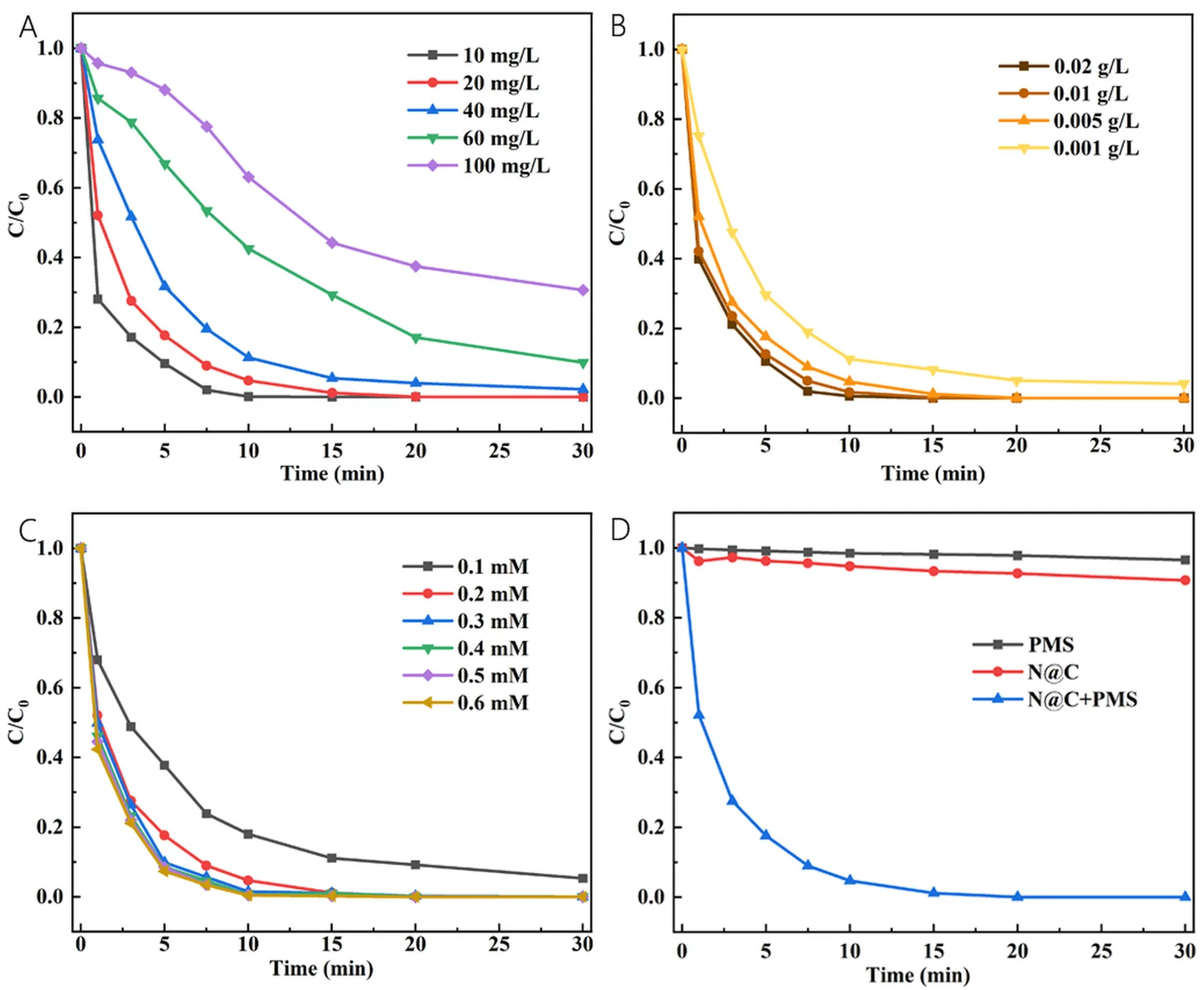
Effect of initial ENR concentration (**A**), N@C loading (**B**), PMS concentration (**C**) on ENR degradation; (**D**) ENR degradation under different systems. [ENR]_0_ = 20 mg L^−1^; [PMS] = 0.2 mM; [N@C] = 5 mg L^−1^; T = 25 °C.

The dependence on [ENR]_0_ was quantified over 10–100 mg L^−1^ (Figure 5A, Table 1). The apparent pseudo-first-order rate constant (*k*_obs_) decreased monotonically as the substrate load increased: *k*_obs_ dropped from 0.6434 min^−1^ at 10 mg L^−1^ to 0.4389, 0.1575, 0.0793, and 0.0442 min^−1^ at 20, 40, 60, and 100 mg L^−1^, with corresponding removal efficiencies: 100.00%, 99.999%, 98.840%, 90.185%, and 69.379%. This inverse relationship reflects a fixed flux of reactive species at a given catalyst/oxidant dose: as more ENR molecules compete for the same steady-state ^1^O_2_ concentration, the per-molecule oxidation probability declines, and higher loadings progressively saturate surface sites, impeding PMS access to catalytic centers. Increasing the N@C dose from 1 to 5 mg L^−1^ raised R% from 95.89% to 99.99%, and at 20 mg L^−1^, 99.99% of ENR was removed within 15 min (Figure 5B); the proportionality between catalyst mass and rate is consistent with a heterogeneous mechanism in which the number of active sites scales with catalyst concentration. The 5 mg L^−1^ N@C therefore represents a practical optimum balancing rate and oxidant economy. As shown in Figure 5C, varying PMS from 0.1 to 0.2 mM increased R% from 94.69% to 99.99%. Further elevating PMS dosage beyond 0.3 mM yielded negligible improvement in ENR removal efficiency, indicating saturation of the catalyst’s activation capacity. The 0.2 mM PMS therefore represents a practical optimum balancing rate and oxidant economy.

Thus, the catalytic activity of N@C toward ENR was evaluated under standard conditions ([ENR]_0_ = 20 mg L^−1^, [PMS] = 0.2 mM, [N@C] = 5 mg L^−1^, 25 °C). The necessity of both catalyst and oxidant was first established by comparing isolated and combined systems. Control experiments (Figure 5D) revealed that PMS alone removed only 3.528% of ENR within 30 min, consistent with the kinetic inertness of unactivated PMS toward fluoroquinolones, and N@C alone removed 9.313% through weak surface adsorption. By contrast, the N@C/PMS system eliminated >99.9% of ENR within 20 min (47.929% at 1 min, 82.390% at 5 min), with a *k*_obs_ of 0.4389 min^−1^. The *k*_obs_ in the combined system exceeds the sum of the two isolated controls by nearly two orders of magnitude, confirming true catalytic activation rather than additive behavior. The *k*_obs_ of the N@C/PMS system is comparable to recently reported metal-carbon and metal-free PMS activation systems for antibiotic degradation (e.g., [17,24,25]) and underscores the efficiency of the metal-free nitrogen-doped carbon design.

#### 3.2.2. Effect of Coexisting Matrix Constituents and pH

**Figure 6:**
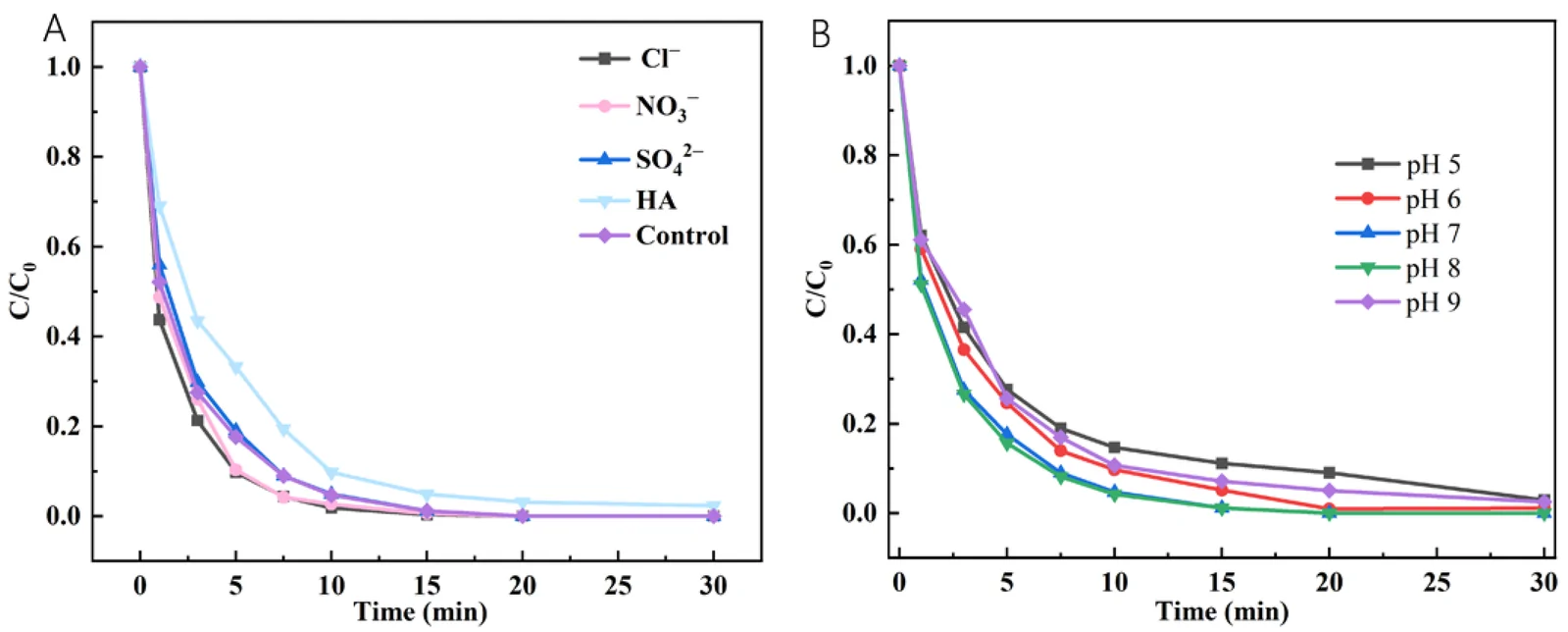
Application potential of N@C. The degradation of ENR in the N@C/PMS system in the presence of different coexisting ions and humic acid (**A**), different initial pH (**B**).

Environmental applicability was assessed by examining the influence of coexisting matrix constituents and solution pH. Among the tested background constituents at 10 mM, Cl^−^, NO_3_^−^, and SO_4_^2−^ caused negligible perturbation (R% ~99.99% for all three), whereas humic acid (HA, 10 mg L^−1^) induced mild inhibition to 97.688% (Figure 6A). This tolerance contrasts sharply with radical-dominated PMS systems, in which such anions competitively scavenge sulfate and hydroxyl radicals; the insensitivity is a direct consequence of the non-radical pathway. HA competes primarily for surface sites without quenching ^1^O_2_ directly. Figure 6B shows that N@C retained high activity across pH 5–9, with the R% of 97.099% (pH 5), 98.880% (pH 6), >99.99% (pH 7), >99.999% (pH 8), and 97.499% (pH 9), indicating a robust near-neutral optimum rather than a narrow pH window [22,26,27].

#### 3.2.3. Cycling Stability, Substrate Scope, and Applicability in Real Water Matrices

**Figure 7:**
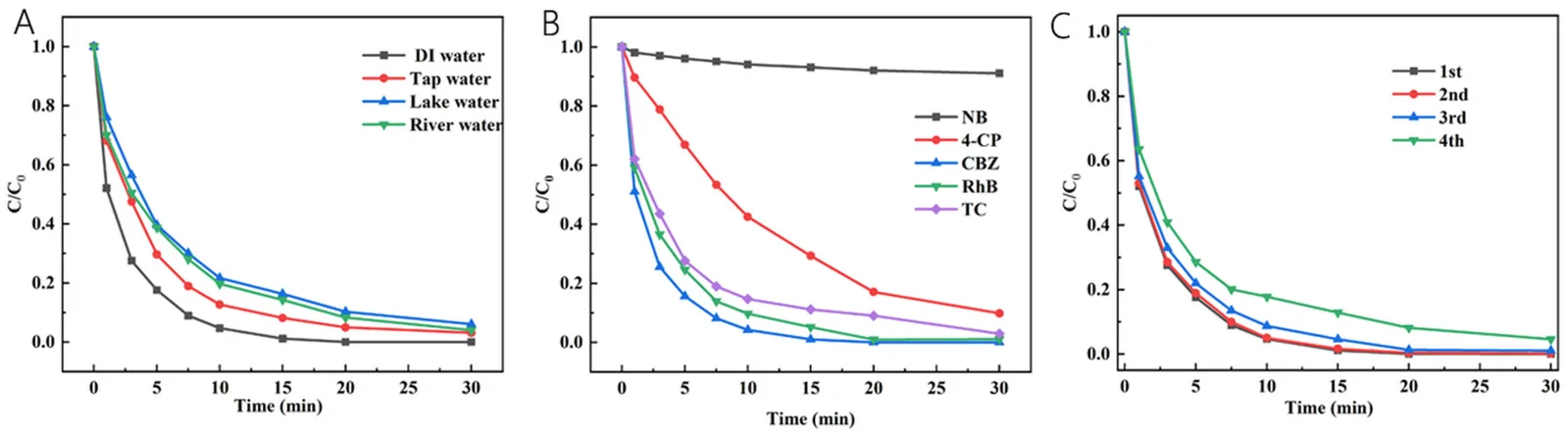
The degradation of ENR in the N@C/PMS system in the different natural waters (**A**), Degradation of multiple pollutants (NB, 4-CP, CBZ, RhB, TC) in the N@C/PMS system (**B**), Reusability of N@C over four consecutive degradation cycles (**C**).

In natural waters (Figure 7A), the system achieved R% of 99.999% (DI water), 96.877% (tap water), 93.977% (lake water), and 95.977% (river water)—the modest reduction in river/lake water reflected natural organic matter competition rather than catalyst deactivation. The substrate scope test revealed pronounced selectivity (Figure 7B): carbamazepine (CBZ, 99.99%), Rhodamine B (RhB, 98.88%), tetracycline (TC, 97.10%), and 4-chlorophenol (4-CP, 90.2%) were readily degraded, whereas nitrobenzene (NB) was barely affected (8.9%). This selective pattern is a hallmark of singlet-oxygen oxidation, as ^1^O_2_ is an electrophilic oxidant whose reactivity depends on electron-rich moieties; non-polar, electron-poor NB is therefore a poor substrate. The selectivity should be recognized as a boundary condition on applicability, defining the range of pollutants amenable to this treatment approach.

Operational durability was evaluated through cycling tests (Figure 7C). The four-cycle test gave R% of 99.999%, 99.812%, 98.972%, and 95.369% (cycles 1–4). The progressive yet limited deactivation arises from the occupancy of active sites by oxidized intermediates, an issue that may be adequately addressed by post-reaction calcination.

### 3.3. Mechanistic Analysis of the Non-Radical Pathway

The results of characterization in Section 3.1 establish a metal-free, nitrogen-rich, defect-abundant carbon architecture, and the performance data (Section 3.2) reveal matrix-tolerant, pH-robust degradation behavior inconsistent with free-radical domination. The reactive oxidant and its generation pathway were therefore identified through chemical quenching, solvent-isotope probing, and ESR spectroscopy.

#### 3.3.1. Radical Quenching Experiments

**Figure 8:**
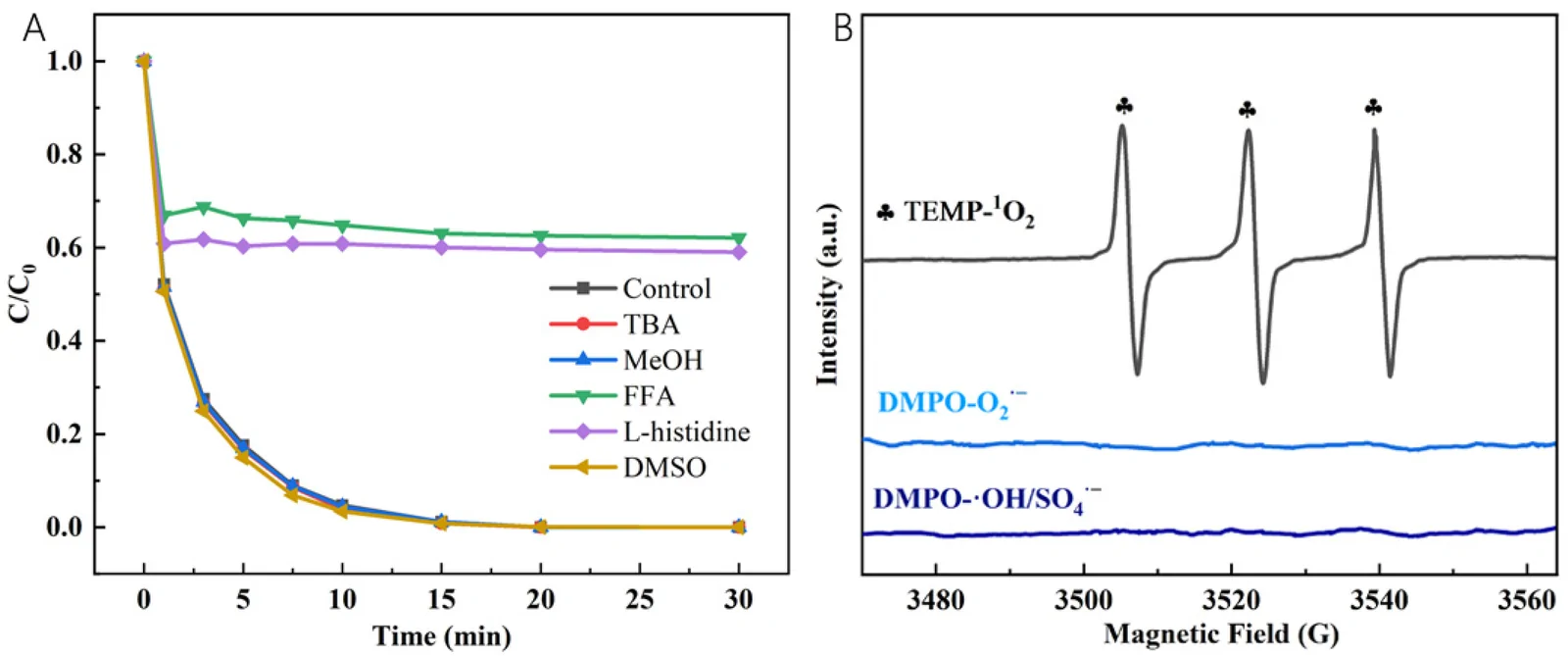
(**A**) Effect of radical scavengers on ENR degradation: TBA (0.1 M), MeOH (0.1 M), FFA (0.02 M), L-histidine (0.02 M) and DMSO (0.1 M). (**B**) ESR spectra obtained with TEMP (for ^1^O_2_) and with DMPO (for •OH, SO_4_•^−^ and •O_2_^−^). Conditions: [ENR]_0_ = 20 mg L^−1^, [PMS] = 0.2 mM, [N@C] = 5 mg L^−1^, T = 25 °C.

Chemical scavenging was used to dissect the contribution of radical versus non-radical species. As shown in Figure 8A, relative to the scavenger-free control (>99.9% removal), spiking TBA (0.1 M, selective for •OH) or MeOH (0.1 M, scavenges both SO_4_•^−^ and •OH) left removal essentially unchanged, both within the scatter of the scavenger-free control; DMSO (0.1 M, for •O_2_^−^) likewise had no measurable effect. In stark contrast, the ^1^O_2_ scavengers FFA (0.02 M) and L-histidine (0.02 M) collapsed removal to 37.903% and 40.993%. The selective, near-total suppression by ^1^O_2_ quenchers—while radical quenchers are ineffective—indicates that ^1^O_2_, not •OH, SO_4_•^−^, or •O_2_^−^, is the principal oxidant. Because FFA and L-histidine are themselves oxidizable and could in principle consume PMS, this evidence is treated with care. The quenchers that suppressed the reaction were applied at a five-fold lower molar excess over PMS (0.02 M, a 100-fold excess) than those that produced no inhibition at all (0.1 M, a 500-fold excess), and non-selective consumption of the oxidant by the reagent would be expected to act in the opposite order; we treat this as a consistency argument rather than as a direct control, and the assignment therefore rests on the independent evidence described below. This assignment was independently supported by a solvent-isotope test: replacing H_2_O with D_2_O (which prolongs ^1^O_2_ lifetime) slightly accelerated degradation (*k*_obs_ = 0.5957 in D_2_O vs. 0.4389 in H_2_O) (Figure S1), the expected direction for a ^1^O_2_-dominated process [21,22,28]. DMSO is not a selective •O_2_^−^ probe, so that result is treated as supporting rather than decisive. Partial rather than complete suppression is the expected behaviour for a competitive quencher in a system where the oxidant is regenerated at the catalyst surface. The residual removal is consistent with the ~9% contribution of adsorption measured in the control experiments and does not require a second reactive species; we do not apportion the remainder quantitatively.

#### 3.3.2. ESR Spectroscopy and the Stoichiometric Probe

Direct detection of ^1^O_2_ was performed by ESR using TEMP as the spin trap (Figure 8B). The N@C/PMS system produced a characteristic 1:1:1 triplet with a hyperfine coupling constant *a_N_* = 17.1 G, consistent with TEMPO in aqueous solution, assigned to the TEMPO adduct formed by specific trapping of ^1^O_2_. This assignment requires care, because TEMPO can also be formed by direct oxidation of TEMP by PMS or by electron transfer from the carbon surface, and all of these routes give the same triplet [29]. In addition, commercial TEMP contains N-oxyl impurities that can bias the measurement [29,30]. The TEMPO signal is therefore treated as supporting evidence rather than as conclusive proof of ^1^O_2_. No DMPO–OH, DMPO–SO_4_ or DMPO–•O_2_^−^ adduct signals were observed under identical conditions, corroborating the quenching results and ruling out a significant radical contribution. Integrating the three lines of evidence—quenching, isotope effect, and ESR—the N@C/PMS system operates predominantly through a ^1^O_2_-dominated non-radical pathway.

**Figure 9:**
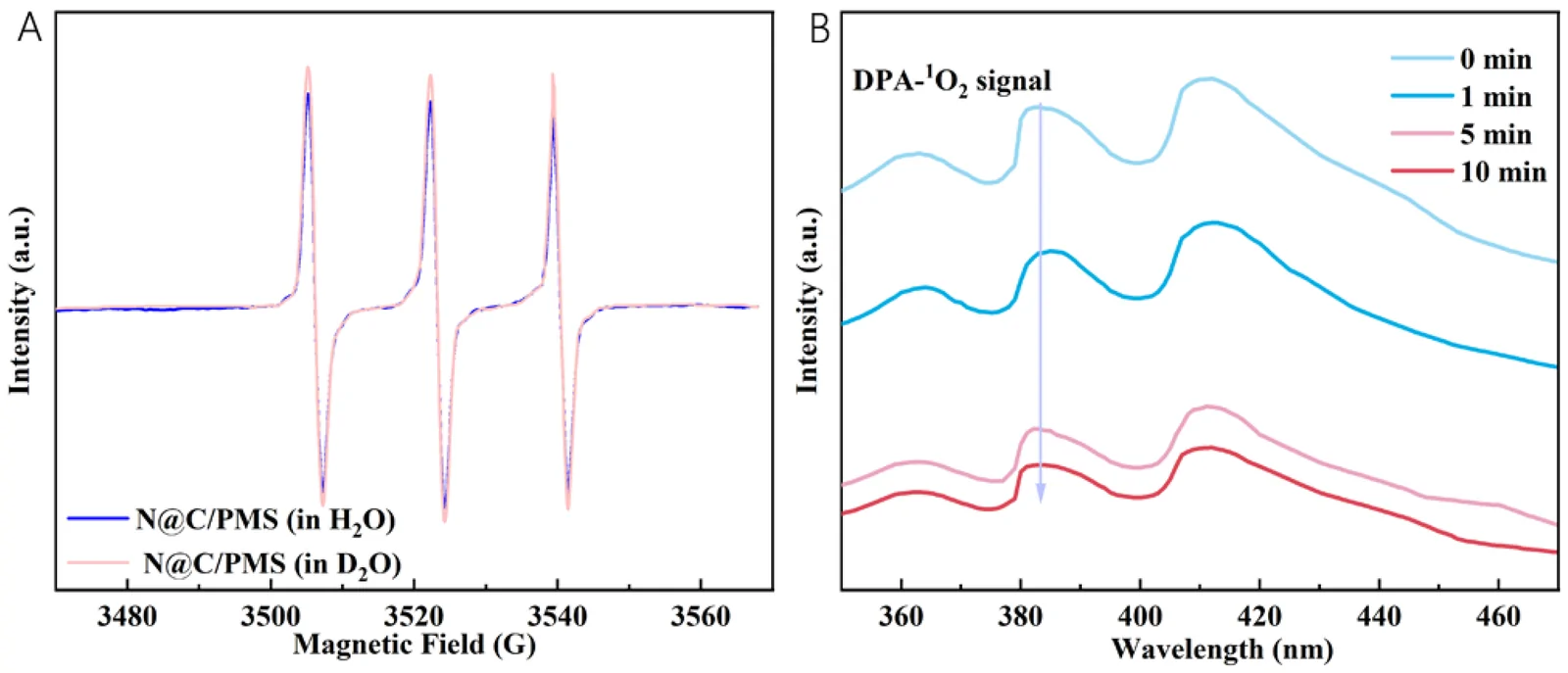
(**A**) ESR spectra of the N@C/PMS system in H_2_O and in D_2_O, with TEMP as the spin trap. (**B**) UV–vis spectra of 9,10-diphenylanthracene (DPA) in the N@C/PMS system, recorded at 0, 1, 5 and 10 min; the decay of the 378 nm band is the DPA–^1^O_2_ signal. Conditions as in Figure 8.

Because the quenching and ESR results exclude a radical pathway and give no indication of mediated electron transfer [31,32], a stoichiometric probe was used to test the ^1^O_2_ pathway directly. DPA reacts with ^1^O_2_ in a 1:1 fashion to form the stable endoperoxide 9,10-diphenylanthracene dioxide (DPAO_2_) [33], so the loss of its 378 nm absorption band is proportional to the amount of ^1^O_2_ consumed. As shown in Figure 9B, this absorbance decreased over time in the N@C/PMS system with no chemical quencher present, which indicates that the ^1^O_2_ generated can reach and oxidize a dissolved substrate; because the consumption of the probe is stoichiometric, this cannot arise from depletion of the oxidant.

#### 3.3.3. Discriminating ^1^O_2_ from Radical and Other Non-Radical Channels

Non-radical oxidation in carbon/PMS systems can proceed through at least three channels: singlet oxygenation by ^1^O_2_, oxidation by surface-bound radicals, and mediated electron transfer through a metastable catalyst–PMS complex [31,32]. The three are frequently conflated because they respond similarly to some conventional quenching tests, and the evidence assembled here is read against that background. The scavenger-free control removed more than 99.9% of the ENR, whereas MeOH and TBA at 0.1 M left removal unchanged, which excludes free SO_4_•^−^ and •OH. FFA and L-histidine at 0.02 M suppressed removal to 38–41%. The D_2_O solvent-isotope effect is positive (Figure 9A). Substrate selectivity is pronounced: electron-poor nitrobenzene is essentially unreactive (8.9%), whereas electron-rich substrates are degraded rapidly. Cl^−^, NO_3_^−^ and SO_4_^2−^ at 10 mM do not measurably scavenge the oxidant (Section 3.2.2). TOC removal reaches only 40% after 30 min, of which 9.3% is attributable to adsorption by the catalyst alone (Section 3.3.5), which is consistent with a mild, selective oxidant. Post-reaction XRD shows that the carbon framework is preserved (Section 3.3.5). A stoichiometric probe reinforces this picture: 9,10-diphenylanthracene (DPA) was bleached at 378 nm in the N@C/PMS system (Figure 9B), and consumption of a probe by ^1^O_2_ cannot be produced by depletion of the oxidant. Two further observations are treated as supporting only: the TEMPO triplet (Section 3.3.2), which is not conclusive on its own, and the absence of any rate change on addition of the superoxide scavenger DMSO. None of the evidence assembled here points to a radical or to an electron-transfer channel, and the site-level interpretation of the ^1^O_2_ pathway is given in Section 3.3.4.

#### 3.3.4. Mechanism of ^1^O_2_ Generation

Two claims made in this section are worth separating. The first concerns the origin of ^1^O_2_. In the related g-C_3_N_4_@glucose system the oxidant is proposed to form through a •O_2_^−^ relay [16]; here, however, the superoxide scavenger DMSO leaves the rate unchanged and no DMPO–•O_2_^−^ adduct is detectable (Section 3.3.1 and Section 3.3.2), so a relay of that kind is not required to account for the ^1^O_2_ flux. The second concerns site equivalence: both graphitic and pyridinic N are present, yet the post-reaction XPS data (Section 3.3.5) show that only the graphitic-N component is displaced during turnover, which is difficult to reconcile with two configurations behaving as interchangeable centres. What follows therefore sets out a mechanism consistent with these two observations rather than a complete site-level proof.

The origin of this pathway is traced to the nitrogen speciation and defect structure elucidated in Section 3.1. PMS activation is initiated at electron-rich carbon atoms adjacent to graphitic-N sites: the lone-pair electrons on graphitic N polarize the neighboring C–N bond, elevate local electron density, and donate electron density into the σ* antibonding orbital of the PMS O–O bond, promoting heterolytic O–O cleavage that selectively yields ^1^O_2_ rather than the radical products of homolytic scission. Pyridinic N complements this as a Lewis-basic edge site that anchors PMS via hydrogen bonding and electrostatic attraction, pre-concentrating the oxidant near the graphitic-N activation centers and lowering the effective barrier. The defect density (I_D_/I_G_ = 1.0511) introduces localized electronic states within the band gap that serve as auxiliary PMS-binding and activation sites, augmenting the overall ^1^O_2_ flux [17,22,34,35,36]. This remains a working model rather than a proven site assignment, because a single catalyst composition cannot isolate the roles of the individual nitrogen configurations; a series of carbons with systematically varied pyridinic/graphitic N ratios would be required to test the descriptor quantitatively (Section 4).

#### 3.3.5. Mineralization and Post-Reaction Structural Stability

Mineralization was monitored by TOC analysis over 30 min at a higher PMS concentration than the standard runs ([PMS] = 1.0 mM; all other conditions as in Section 2.5). TOC removal reached 40%, whereas the parallel control without PMS removed 9.3% (Figure S2), so about 31% of the organic carbon was genuinely mineralized. This is far below the more than 99.9% removal of the parent compound measured by HPLC, and the two time courses are widely separated: at 20 min, when more than 99.9% of the parent compound had already been removed, total TOC removal was still only 15%. ENR is therefore attacked rapidly but mineralized only slowly: most of the oxidant is consumed in non-mineralizing steps while the parent compound remains, and mineralization accelerates only once the parent has been depleted, so that TOC removal continues to rise throughout the 30 min window rather than levelling off. This behaviour is characteristic of selective, electrophilic oxidation by ^1^O_2_, which converts the parent molecule into partially oxidized products rather than fully to CO_2_; the same gap would be difficult to reconcile with radical-dominated chemistry, because SO_4_•^−^ and •OH are comparatively non-selective. The removal measured in the control without PMS (9.3%) agrees closely with the 9.313% ENR removal determined for the catalyst alone by HPLC, which supports attributing this contribution to adsorption rather than to oxidation. Partial mineralization also defines the practical limitation of the system, since an N@C/PMS polishing step will attenuate ENR but will not, on its own, mineralize it.

**Figure 10:**
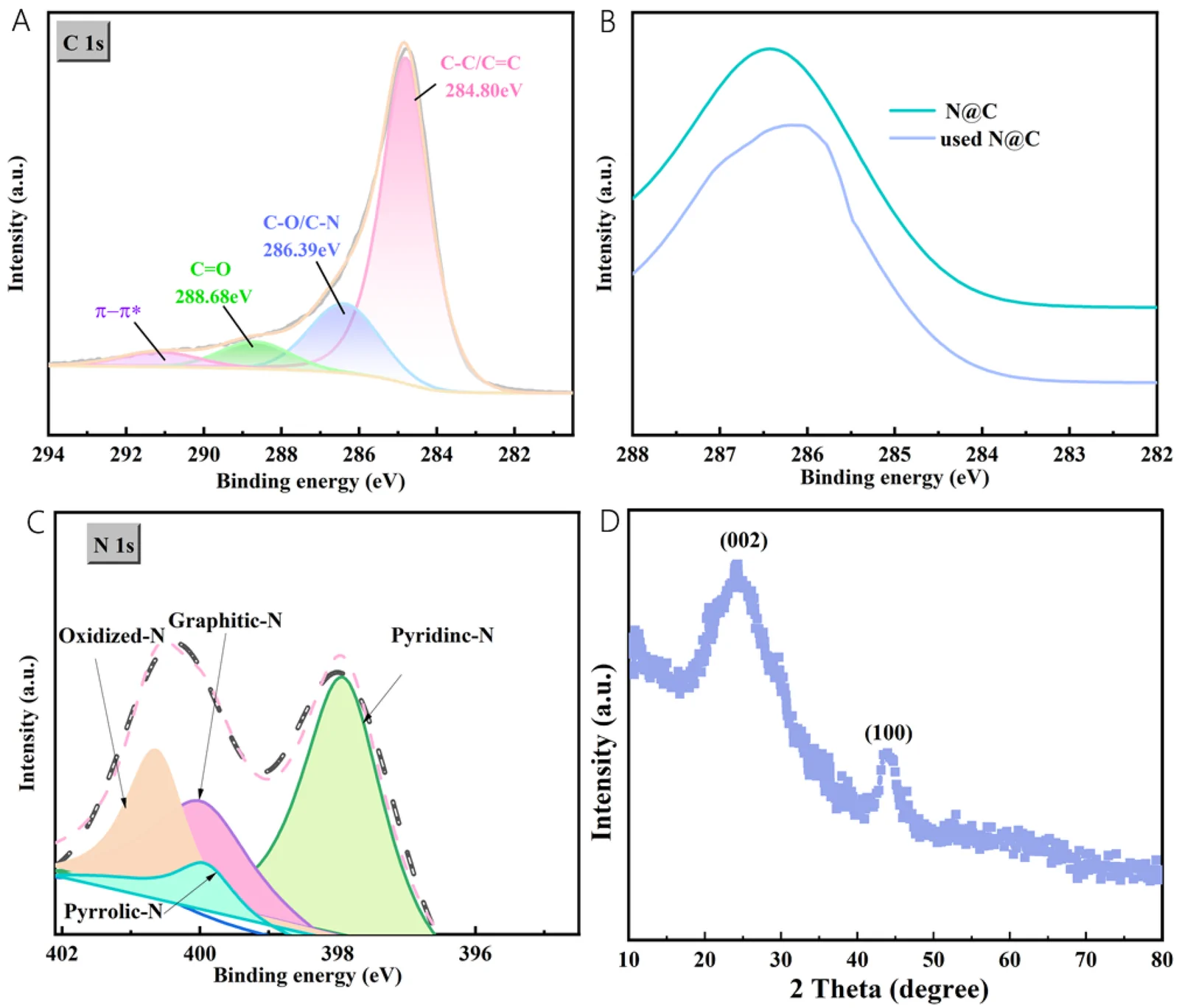
(**A**) High-resolution C 1s spectrum of the used N@C. (**B**) C 1s spectra of N@C and of the used N@C, normalized and overlaid. (**C**) High-resolution N 1s spectrum of the used N@C. (**D**) XRD patterns of the used N@C.

The catalyst recovered after four degradation cycles was examined by XPS and XRD (Figure 10). Its C 1s envelope retained the same four components as the fresh catalyst, but the C–N/C–O component was displaced by 0.4 eV towards lower binding energy (Figure 10A,B). A smaller displacement was found in the N 1s region: the component assigned to graphitic N moved to lower binding energy by 0.15 eV, whereas the pyridinic-N and pyrrolic-N components were unchanged within the experimental resolution (Figure 10C). Because a binding-energy shift reports a change in the local electronic environment rather than a change in the amount of a species, the two configurations are evidently not perturbed to the same degree during turnover: the one that responds is the configuration identified above as the activation site, whereas the other behaves as a spectator [11]. The XRD patterns of the fresh and used catalysts (Figure 10D) were essentially identical, so the carbon framework was preserved within the detection limit of the technique. Deactivation is therefore attributable to occupancy of the active sites by oxidized intermediates rather than to structural collapse, consistent with the limited activity loss observed over four consecutive cycles.

### 3.4. Comparison with Prior Studies

Table 2 places N@C in the context of representative PMS-based antibiotic-degradation studies. The comparison highlights that metal-free, nitrogen-doped carbons activating PMS via ^1^O_2_ offer a distinct advantage over metal-based radical systems in avoiding metal leaching and matrix scavenging. The g-C_3_N_4_-derived route delivers a high surface area (578.6 m^2^ g^−1^) and stable cycling that compare favorably with related carbon catalysts. The closest prior work, the g-C_3_N_4_@glucose-derived carbon reported by Zhang et al. [16], also activates PMS through ^1^O_2_, but proposes that ^1^O_2_ arises from a superoxide (•O_2_^−^) intermediate and treats graphitic and pyridinic N as co-equal catalytic sites. In the present system a •O_2_^−^ probe has no measurable effect on the rate, and the results instead support direct heterolytic O–O cleavage of PMS at graphitic-N-adjacent carbon, with pyridinic N acting as an anchoring site. The descriptor proposed for the two systems therefore also differs: ref. [16] correlates activity with the absolute amounts of graphitic N and pyridinic N, whereas the present data point to the relative population of the two configurations.

## 4. Conclusions

A metal-free nitrogen-doped carbon catalyst (N@C) was successfully fabricated from g-C_3_N_4_ and glucose via hydrothermal compositing and high-temperature carbonization. N@C possessed a hierarchically porous architecture with a high specific surface area of 578.6 m^2^ g^−1^ and a nitrogen content of 10.2 at%, and achieved over 99.9% ENR removal within 20 min under standard conditions ([ENR]_0_ = 20 mg L^−1^, [PMS] = 0.2 mM, [N@C] = 5 mg L^−1^, 25 °C) with a *k*_obs_ of 0.4389 min^−1^. Mechanistic investigations indicate that degradation proceeds predominantly through a non-radical pathway dominated by ^1^O_2_, initiated by heterolytic cleavage of the PMS peroxide bond at graphitic nitrogen sites, while pyridinic nitrogen acts as a Lewis-basic anchor that pre-concentrates PMS near the activation centers. The system exhibited strong tolerance to common water-matrix constituents (Cl^−^, NO_3_^−^, SO_4_^2−^) and maintained above 93% removal efficiency in authentic water matrices, with the catalyst retaining over 95% of its initial activity after four consecutive reuse cycles. TOC removal reached 40% within 30 min, of which 9.3% was attributable to adsorption by the catalyst alone; the parent compound was therefore converted largely into partially oxidized intermediates rather than fully mineralized, which is consistent with a mild, selective oxidant. Post-reaction XPS and XRD showed that the carbon framework was preserved and that only the graphitic-N component was perturbed during turnover.

These results advance the structure–activity understanding of metal-free carbon catalysts by clarifying the distinct, cooperative roles of nitrogen species in PMS activation. Rather than attributing non-radical activation solely to carbon defects, the present results support a nitrogen-speciation model in which the relative abundance of pyridinic and graphitic nitrogen—rather than total nitrogen content—is proposed to govern singlet-oxygen productivity. The resulting design principle, that nitrogen configuration can be tuned to steer the reactive-oxygen pathway away from radical-dominated, matrix-sensitive chemistry, offers a mechanistic basis for the rational construction of metal-free persulfate activators. Because N@C operates without metal loading, it eliminates the risk of metal-ion leaching that constrains cobalt- and iron-based activators, and its ^1^O_2_-dominated pathway offers practical promise for treating refractory fluoroquinolone wastewaters. Testing this descriptor quantitatively requires a series of catalysts with systematically varied nitrogen speciation, which is identified as a priority for future work.

Future research perspectives are proposed as follows: (i) resolving transformation pathways through LC-MS/MS coupled with TOC and ecotoxicity assays, and testing the proposed nitrogen-speciation descriptor across a series of catalysts with systematically varied pyridinic/graphitic N ratios; (ii) evaluating long-term, flow-through stability and regeneration under field-relevant matrices; (iii) employing operando spectroscopy and DFT calculations to directly probe the PMS–carbon interface and quantify site-specific contributions; (iv) exploring tandem radical/non-radical designs to broaden the pollutant scope beyond the electron-rich substrates favored by ^1^O_2_ chemistry; and (v) integrating N@C with semiconductor light absorbers to construct metal-free heterojunction photocatalysts that couple photocatalytic charge separation with PMS activation.

## Figures and Tables

**Table 1:** *k*_obs_ for ENR degradation at varying ENR initial concentrations ([PMS] = 0.2 mM, [N@C] = 5 mg L^−1^, 25 °C).

[ENR]_0_ (mg L^−1^)	*k*_obs_ (min^−1^)	R^2^	R%
10	0.6434	0.915	100.00
20	0.4389	0.964	99.999
40	0.1575	0.928	98.840
60	0.0793	0.993	90.185
100	0.0442	0.956	69.379

**Table 2:** Comparison of N@C with representative PMS-based degradation studies.

Reference	Activator/Material	Target Pollutant	Dominant ROS/Mechanism	Notable Feature
This work	N@C (g-C_3_N_4_ + glucose, 900 °C, metal-free)	Enrofloxacin	^1^O_2_ non-radical	*k*_obs_ = 0.4389 min^−1^; >99.9%@20 min; 4-cycle > 95%
[1] Lin et al., 2025	N-CDs/g-C_3_N_4_/MIL-101(Fe) membrane	Enrofloxacin	PMS + photocatalysis	Contains Fe; hybrid process
[2] Wang et al., 2024	CoFe_2_O_4_@biochar	Enrofloxacin	Radical (Co-based)	Metal leaching risk; requires separation
[10] Yun et al., 2018	N-doped carbon/various	Organic dyes	^1^O_2_ vs. electron transfer	Discriminated radical vs. non-radical mechanisms
[11] Gao et al., 2020	N-doped carbon (metal-free)	Phenol/antibiotics	^1^O_2_ non-radical	Metal-free; electron-deficient carbon
[13] Wang et al., 2022	Intrinsic-defect carbon	Tetracycline	Defect-dominated	Inherent carbon defects
[14] Wang et al., 2022	Ring-defect-rich pyridinic-N graphene	Tetracycline	Defect/pyridinic-N	Defect engineering for TC
[16] Zhang et al., 2023	g-C_3_N_4_@glucose-derived carbon (metal-free, N-tunable)	Levofloxacin	^1^O_2_ from •O_2_^−^; graphitic + pyridinic N	Closest prior art; N content tuned by glucose/g-C_3_N_4_ ratio
[17] Xiong et al., 2025	N/O dual-doped porous carbon (g-C_3_N_4_-derived)	Tetracycline	^1^O_2_ + O_2_•^−^	Metal-free; graphitic/pyridinic N + C=O synergy
[24] Niu et al., 2024	Cu/MnO carbon framework	Enrofloxacin	^1^O_2_ + O_2_•^−^	Metal-containing; bimetallic redox couple
[25] Xiao et al., 2025	Cu–Co–O/g-C_3_N_4_	Sulfamethoxazole	^1^O_2_ dominated (minor radical)	Metal-containing; g-C_3_N_4_ support
[34] Zheng et al., 2024	Fe-N-C (MOF-gel derived)	Amoxicillin	^1^O_2_ dominated	Magnetic recyclable; lattice-oxygen cleavage

## Data Availability

The original contributions presented in this study are included in the article/Supplementary Material. Further inquiries can be directed to the corresponding author.

## Supplementary Materials

The following supporting information can be downloaded at: https://www.mdpi.com/article/10.3390/nano16211427/s1, Figure S1 (Effects of D2O on ENR degradation), Figure S2 (TOC removal of the N@C/PMS system (1 mM PMS) and of the N@C-only control), and Supplementary Text S1 (DPA probe: measurement protocol) can be found online.
