# Supplementary material for: Metal-Free Nitrogen-Doped Carbon Derived from g-C3N4/Glucose Composites for Singlet Oxygen-Dominated PMS Activation Toward Enrofloxacin Degradation
Source: Nanomaterials (Basel). 2026 Nov 5;16(21):1427. doi: 10.3390/nano16211427 (PMC13764891; doi:10.3390/nano16211427)
Supplement: Supplementary file 1 [file nanomaterials-16-01427-s001.zip › nanomaterials-4565318-supplementary.pdf]

Supporting information

Metal-Free Nitrogen-Doped Carbon Derived from g-C_3N_4/Glucose Composites for Singlet Oxygen-Dominated PMS Activation Toward Enrofloxacin Degradation

Peilun Wang [1,2,†], Lei Wang [1,†], Xinyu Zhang [3], Yinlong Kuang [4], Shuming Liu [1], Le Cui [1], Yixue Xu [1] and Qiufang Yao [1,2,*]

[1] College of Advanced Materials Engineering, Jiaxing Nanhu University, Jiaxing 314001, China
[2] Yangtze River Delta Institute of Circular Economy Technology, Jiaxing, 314001, China
[3] Shanghai Chengtou Water Group Co., Ltd., Shanghai 200092, China
[4] College of Civil Engineering and Architecture, Zhejiang University, Hangzhou 310058, China
* Correspondence: qfyao@jxnhu.edu.cn
† These authors contributed equally to this work.

Supplementary Text S1. DPA probe: measurement protocol.

A DPA stock solution was prepared by dissolving 10 mg of DPA in 10 mL of DMSO (1 mg mL^{-1}). For each run, 100 μL of this stock was diluted with 1.8 mL of water, and the reaction was initiated by adding 1.6 mL of the N@C/PMS suspension. Samples were taken at the chosen times and passed immediately through a 0.22 μm membrane; removing the catalyst stops the reaction, because no further $^1O_2$ can be generated without it, and any $^1O_2$ already present in solution decays within a few microseconds. Because no quencher was added, the filtrate could be measured directly. DPA was quantified from its absorbance at 378 nm on a UV–vis spectrophotometer; the decomposition rate was obtained from the decrease in that absorbance.

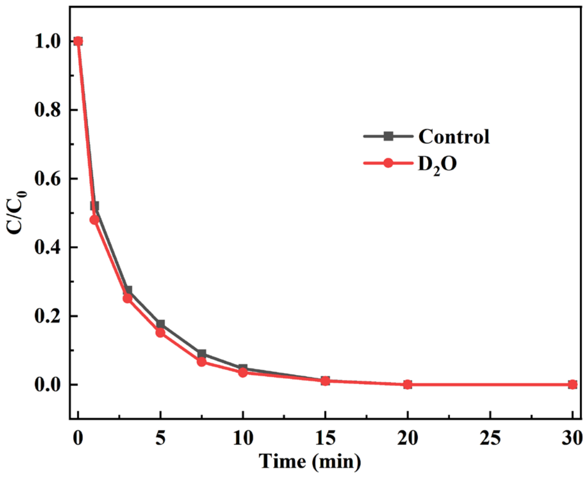

Figure S1. Effects of D_2O on ENR degradation.

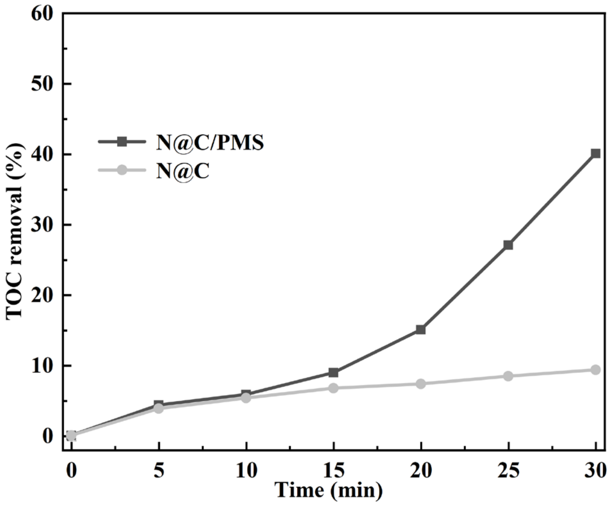

Figure S2. TOC removal of the N@C/PMS system (1 mM PMS) and of the N@C-only control.
